# The renal transport of hippurate and protein‐bound solutes

**DOI:** 10.14814/phy2.14349

**Published:** 2020-02-25

**Authors:** Rohit Kumar, Avinash Adiga, Joshua Novack, Alex Etinger, Lawrence Chinitz, James Slater, Henriette de Loor, Bjorn Meijers, Robert S. Holzman, Jerome Lowenstein

**Affiliations:** ^1^ NYU School of Medicine New York NY USA; ^2^ Nephrology and Renal Transplantation Research Group Department of Microbiology, Immunology and Transplantation KU Leuven Leuven Belgium; ^3^ Department of Nephrology and Renal Transplantation University Hospitals Leuven Leuven Belgium

**Keywords:** effective renal plasma flow, hippurate clearance, protein‐bound solutes, renal hemodynamics

## Abstract

Measurement of the concentration of hippurate in the inferior vena cava and renal blood samples performed in 13 subjects with normal or near‐normal serum creatinine concentrations confirmed the prediction that endogenous hippurate was cleared on a single pass through the kidney with the same avidity as that reported for infused para‐amino hippurate. This suggests that a timed urine collection without infusion would provide a measure of effective renal plasma flow. Comparison of the arteriovenous concentration differences for a panel of protein‐bound solutes identified solutes that were secreted by the renal tubule and solutes that were subjected to tubular reabsorption.

## Graphical abstract

The renal extraction of hippurate suggests that it can serve for the estimation of effective renal plasma flow without the need for infusion. The free (unbound) fraction of protein‐bound solutes are filtered at the glomerulus. Bound solutes are transported across the renal vascular bed, by organic anion transporters.

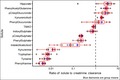

## 1 BACKGROUND

It has been known since the early part of the twentieth century that some small molecules are bound to plasma proteins. Pharmacologists viewed the possible competition of drugs for such binding as possible determinants of drug effect and excretion (Bowmer & Lindup, 1982). Renal physiologists recognized that although para‐amino hippurate (PAH) was protein‐bound, the clearance of PAH provided an estimate of renal plasma flow (RPF) (Smith). For most of the latter half of the twentieth century, the glomerular filtration rate (GFR) and effective renal plasma flow (ERPF) were assessed by measuring the plasma clearances of inulin and PAH (Smith, Finkelstein, Aliminosa, Crawford, & Graber, 1945). These measurements were not widely available because their determination required constant infusion, timed urine collection, and a specialized laboratory. However, a number of clinical disorders were identified in which the filtration fraction (FF), the ratio of the GFR to ERPF, varied considerably from that found in healthy persons. Hypertension and heart failure were characterized by elevated FF (Gómez et al., 1965). Acute glomerulonephritis (Earle, Farber, Alexander, & Pellegrino, 1951) and azotemic patients with severe minimal change nephrotic syndrome (Lowenstein, Schacht, & Baldwin, 1981) exhibited markedly reduced FF.

Since 1999, the estimation of GFR has come to be based on the measurement of serum creatinine concentration (eGFR) and no longer requires urine sampling (Levey et al., 1999). Despite the evidence that RPF may vary independently of GFR, RPF is no longer measured and renal function has come to be estimated solely by eGFR. In 2016, we (Lowenstein & Grantham, 2016) suggested that the clearance of endogenous hippurate might provide a measure of ERPF. We recognized that utilizing hippurate clearance as a measure of RPF would require verification that hippurate was transported by the renal tubule to the same extent as PAH, the highly efficient clearance of which enabled estimation of the ERPF using the Fick principle (Fick, 1855). At a low plasma concentration, roughly 90% of PAH is removed from blood entering the renal artery and the concentration of PAH in the renal vein (RV) approaches zero. Determination of ERPF reduces to urinary excretion of PAH divided by the concentration of PAH in a peripheral vein (Smith et al., 1945). We undertook to measure the extraction ratio, the fraction of hippurate removed from the blood during transit through the kidney, in order to confirm that the clearance of hippurate could provide a method for determining ERPF.

Our interest in the renal clearance of hippurate comes at a time at which there is considerable interest in the role of protein‐bound uremic retention solutes in cardiovascular disease in patients undergoing maintenance hemo‐ or peritoneal dialysis (United States Renal Data System). The collection of a renal venous blood sample provided us with the opportunity to examine the renal transport of a panel of protein‐bound solutes. The recognition that protein‐bound solutes might be of importance in the pathophysiology of uremia followed the identification of specific organic anion transporter (OAT 1) in the proximal renal tubule (VanWert, Gionfriddo, & Sweet, 2010) and the recognition that basolateral transport of a wide variety of solutes, most notably protein‐bound organic anions (Nigam et al., 2015) was mediated by OAT 1 and the related OAT 3. These findings were soon followed by a report by the EuTox group (Vanholder et al., 2003) that identified more than 100 solutes that exhibited increased concentration in the serum of patients with impaired renal function. While roughly one‐half of these solutes are small, water soluble, readily dialyzable solutes such as urea and creatinine, roughly 25 are small molecules that are partially or highly protein bound. These bound solutes are too large to be removed by glomerular filtration. Many of these protein‐bound solutes are transported across the basolateral and apical membranes of the renal tubule by organic anion and organic cation transporters on proximal renal tubular cells (VanWert et al., 2010). The mechanism and magnitude of renal tubular transport of protein‐bound solutes may be important determinants of renal excretion of these solutes and of significance when renal tubular function is disturbed or the nephron mass reduced. Our studies of transport of hippurate across the renal vascular bed provided us with the opportunity to examine the transport of a panel of protein‐bound solutes across the renal vascular bed of patients with apparently normal renal function as assessed by the serum creatinine level.

## 2 METHODS

Studies were carried out in 13 subjects. Four subjects with hypertrophic cardiomyopathy were studied during preoperative right heart catheterization. They ranged in age from 43 to 73 years and had recorded serum creatinine concentrations between 0.9 and 1.0 mg/dl. Nine subjects with atrial fibrillation were studied while undergoing electrophysiologic studies; they ranged in age from 38 to 81. Serum creatinine was <1.0 in six subjects and 1.16, 1.28, and 1.42 mg/dl in three subjects. All patients undergoing electrophysiologic studies had atrial fibrillation; 7 were receiving apixaban, 8 were receiving beta‐blockers, 2 were receiving ACE inhibitors. One subject with hypertrophic cardiomyopathy was receiving a diuretic. None of the patients manifested signs of congestive heart failure. A voided urine sample was collected 1–3 hr before the RV catheterization. A timed urine specimen was not collected as we deemed it likely that RPF would be effected by the often prolonged study and medications administered during the procedures.

In all subjects, immediately following femoral vein access, a sampling catheter was advanced into the right RV and a blood sample obtained. The catheter was then withdrawn into the inferior vena cava (IVC) below the level of the RV and a second blood sample was obtained.

A panel of 14 protein‐bound solutes including hippurate was measured utilizing MS‐HPLC (de Loor et al., 2016). Nine solutes, hippurate, indole‐3‐ acetic acid, indoxyl sulphate, kynurenic acid, p‐cresyl glucuronide, p‐cresyl sulphate, phenylacetylglutamine, phenyl glucuronide, and phenyl sulphate are anions at physiologic pH (7.4). Four, kynurenine, phenyl alanine, tryptophan, and tyrosine have no net charge at physiologic pH. Trimethylamine oxide is a cation.

Creatinine (Cr) concentration in plasma (P) and urine (U) was measured by a colorimetric technique (Ahmed, 2016).

### 2.1 Computations and analysis

All data were received in Excel Spreadsheets and imported into the R statistical environment for processing. All concentrations were expressed as µM/ml.

Extraction ratios were computed as

$$\frac{{\text{IVC}}_{\text{Solute}}-{\text{RV}}_{\text{Solute}}}{{\text{IVC}}_{\text{Solute}}}$$

We examined whether each solute was secreted or reabsorbed in the nephron by computing the ratio of its clearance to that of creatinine.

$$\frac{{\text{Urine}}_{\text{Solute}}/{\text{Plasma}}_{\text{Solute}}}{{\text{Urine}}_{\text{Creat}}/{\text{Plasma}}_{\text{Creat}}}$$

The fraction of plasma that was filtered was computed from the ratio of GFR to RPF as estimated from the ratio of clearances of creatinine and hippurate.

$$\frac{{\text{Urine}}_{\text{Creat}}/{\text{Plasma}}_{\text{Creat}}}{{\text{Urine}}_{\text{Hippurate}}/{\text{Plasma}}_{\text{Hippurate}}}$$

The removal of solute by glomerular filtration was estimated by assuming that the amount of solute filtered from 1 ml of plasma was equal to the concentration in plasma times the unbound fraction of solute times the ratio of GFR to RPF.

$$\begin{array}{c} {\text{Filtrate}}_{\text{Solute}}\left(\frac{\mu M}{\text{ml}}\right)={\text{RA}}_{\text{Solute}}\left(\frac{\mu M}{\text{ml}}\right)\\ \;\;\;\;\times \text{Fraction Unbound}\times \text{Ratio GFR}/\text{RPF} \end{array}$$

Postglomerular changes (net of the processes of secretion, reabsorption, or metabolism) of all solutes were further quantitated by subtracting the amount of solute filtered from each ml of renal plasma from the amount removed per ml (the *A* − *V* difference).

$$\begin{array}{c} {\text{Sol}}_{\text{Net addition or removal}}\left(\frac{\mu M}{\text{ml}}\right)=\text{Renal}\;A-V\;\text{difference}\left(\frac{\mu M}{\text{ml}}\right)\\ \;\;\;\;\;\;-{\text{Filtrate}}_{\text{Solute}}\left(\frac{\mu M}{\text{ml}}\right). \end{array}$$

## 3 RESULTS

### 3.1 Clearance ratios, extraction ratios and protein binding

Solutes with the highest renal extraction ratios (Hippurate = 0.49, phenyl acetyl glutamine = 0.43, p‐cresyl glucuronide = 0.38, phenyl glucuronide = 0.36 and Kynurenic Acid = 0.32) exhibited the highest ratios of solute clearance to creatinine clearance, exceeding 1, consistent with the tubular secretion of these solutes. Among these 5 solutes, the degree of protein binding differed widely and among all 14 solutes there was no consistent relationship between the fraction bound and either extraction or clearance ratio.

**Figure 1:**
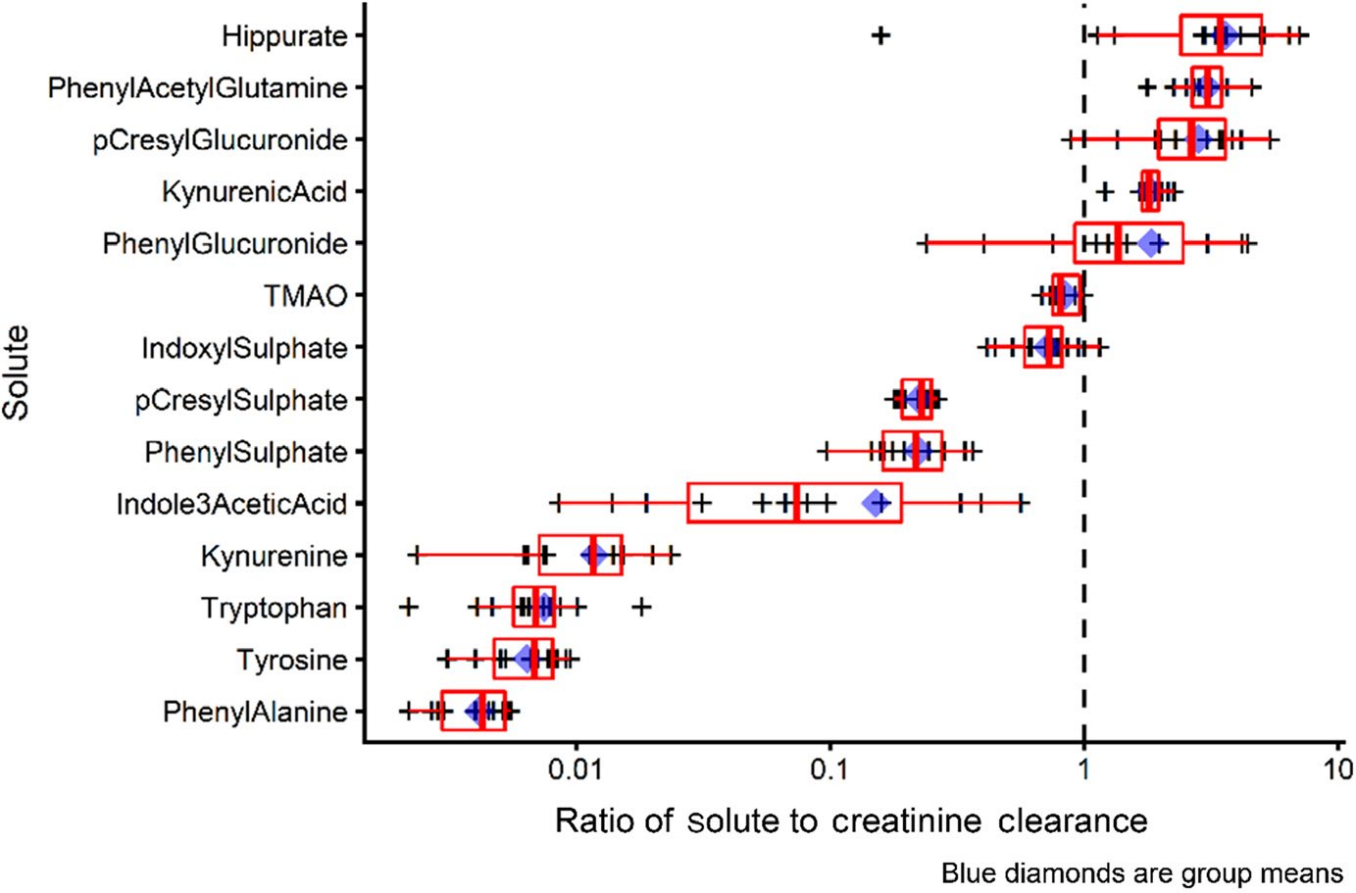
Ratio of solute clearance to creatinine clearance

Among the 12 patients with urine samples available, the mean ratio of solute clearance to creatinine clearance was >1 (range 3.6–1.8) for hippurate, phenyl acetyl glutamine, p‐cresyl glucuronide, kynurenic acid, and phenyl glucuronide confirming their tubular secretion (Figure 1 and Table 1). Mean clearance ratios for the anionic solutes indoxyl sulfate, p‐cresyl sulfate, and phenyl sulfate exceeded 0.1. Given the fact that these solutes were highly bound, the observed clearance ratios, provide evidence of tubular secretion. A solute that is 90% bound, filtered, and neither secreted nor reabsorbed would have a clearance ratio of ~0.1. The conclusion that indoxyl sulfate, p‐ cresyl sulfate, and phenyl sulfate were secreted was confirmed by analysis of the net change due to tubular secretion (Figure 4). Clearance ratios, <0.01, were observed for tryptophan, tyrosine, phenylalanine, and kynurenine, consistent with the known renal tubular reabsorption of these small uncharged solutes, again confirmed in Figure 4.

**Table 1:** Protein binding, renal extraction ratios, and ratios of clearance to creatinine clearance for 14 uremic solutes

Solute	Fraction bound^a^	Renal extraction ratio	Ratio of solute clearance to creatinine clearance
Kynurenic acid	0.94 ± 0.02	0.32 ± 0.21	1.82 ± 0.27
p‐Cresyl sulphate	0.89 ± 0.06	0.07 ± 0.12	0.22 ± 0.03
Phenyl sulphate	0.89 ± 0.06	0.10 ± 0.13	0.22 ± 0.08
Indoxyl sulphate	0.86 ± 0.06	0.12 ± 0.15	0.72 ± 0.21
Tryptophan	0.69 ± 0.16	0.04 ± 0.08	0.011 ± 0.004
Kynurenine	0.63 ± 0.19	0.24 ± 0.18	0.01 ± 0.01
Indole‐3‐acetic acid	0.50 ± 0.17	0.05 ± 0.11	0.15 ± 0.18
Hippurate	0.40 ± 0.08	0.49 ± 0.31	3.59 ± 2.10
p‐Cresyl glucuronide	0.12 ± 0.04	0.38 ± 0.36	2.81 ± 1.30
Phenylalanine	0.03 ± 0.05	0.08 ± 0.08	0.004 ± 0.001
TMAO	0.03 ± 0.04	0.18 ± 0.14	0.84 ± 0.12
Phenyl glucuronide	0.01 ± 0.02	0.36 ± 0.28	2.40 ± 1.61
Phenyl acetyl glutamine	0.01 ± 0.02	0.43 ± 0.32	3.05 ± 0.73
Tyrosine	0	0.01 ± 0.10	0.006 ± 0.002

a The unbound fraction, measured in plasma ultrafiltrate, was derived from a prior study in subjects with ESRD (Etinger et al., 2018). As reported by Deltombe et al. binding of hippurate averaged 34% in healthy controls and 39% in subjects with ESRD receiving hemodialysis (Deltombe et al., 2015). Similarly, the binding of Indoxyl sulfate and p‐cresyl sulfate remained >90% in subjects with ESRD (Deltombe et al., 2015).

### 3.2 Renal extraction of hippuric acid

The fraction of hippurate removed from plasma in a single pass through the kidneys varied from over 0.90 in two subjects to 0 in two subjects.

**Figure 2:**
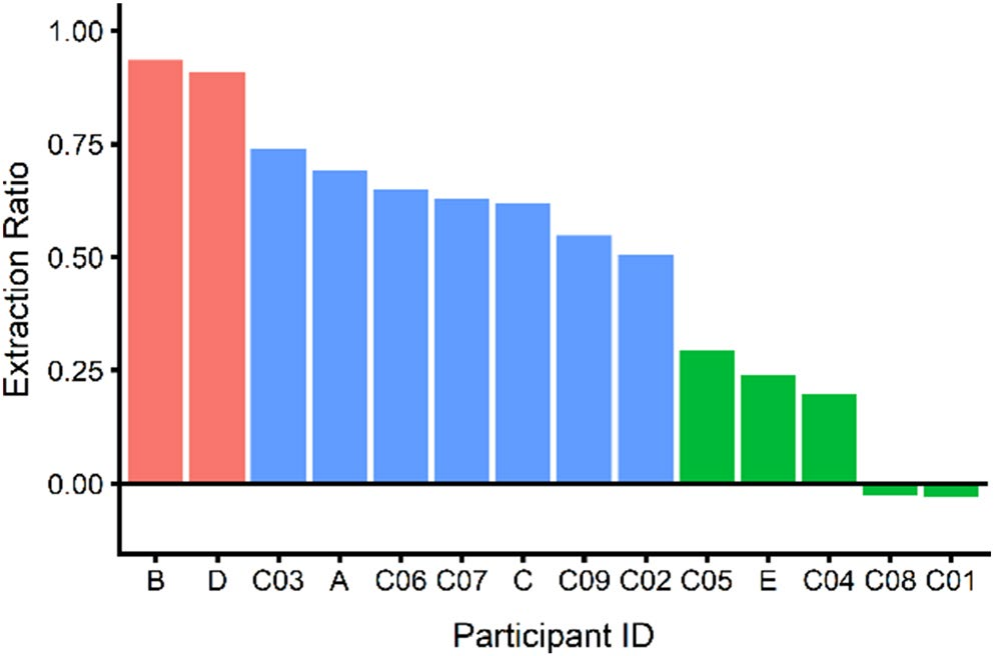
Extraction ratios for hippurate

When ranked, the ratios fell into three groups (Figure 2). Two subjects had ratios over 0.9, clearly in the range seen with p‐aminohippurate in normal subjects. Seven subjects had extraction ratios between 0.74 and 0.50. These ratios are likely to be true values for extraction, possibly reflecting reduced extraction due to reductions in renal function, competitive or noncompetitive inhibition of OAT transport, or intrarenal shunting of blood. Five subjects had extraction ratios below 0.30. We believe these ratios reflect the inadvertent admixture of IV and IVC blood in CO5, E, and CO4. RV‐IVC difference (and therefore the extraction ratio) was zero, in CO8 and CO1; it is likely that these RV samples actually represented IVC sampling.

### 3.3 Hippurate clearance and glomerular filtration

**Figure 3:**
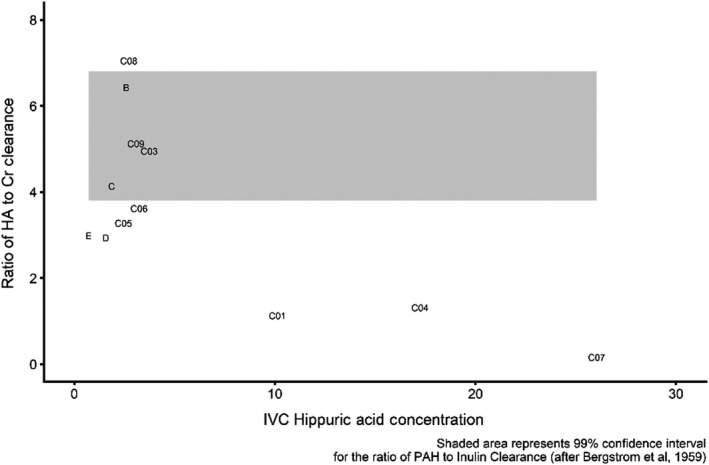
Clearance ratios for hippuric acid plotted against hippuric acid concentration

Shown in Figure 3, as a shaded area, is the 99% confidence interval for the ratio of PAH clearance to inulin clearance based on the data reported by Bergström et al. (1959). We take the overlap between our data and the shaded area as evidence that hippurate clearance may serve as an alternative to clearance of PAH.

Among the nine subjects whose hippurate concentrations were <10 µM/ml, the ratio of hippurate to creatinine clearance ranged from 2.9 to 7.0. All nine had creatinine levels under 1.38 mg/dl. This is in accord with the early observation that RPF can vary widely and independently from GFR (Earle et al., 1951; Lowenstein et al., 1981).

Of note, however, the clearance ratio of hippurate to creatinine was notably lower than the range defined by Bergstrom's data in three of our subjects (CO1, C04, and CO7). These subjects, whose serum creatinine concentrations were 1.28, 0.72, and 0.84 mg/dl, respectively, had plasma concentrations of hippurate substantially greater than those in the rest of the study group. It is known that plasma hippurate concentration is influenced by dietary factors and by gut microbiome composition (Pallister et al., 2017). It seems likely that the observed reduced hippurate clearance seen in these three patients is related to their higher plasma hippurate concentration. It is well established that while the excretion of PAH increases with increased plasma concentration, the extraction ratio (E_PAH_) declines. While the infusion rate of p‐amino hippurate was adjusted to maintain the low concentration required for the measurement of PAH clearance as a measure of RPF, variations in the gut production of hippurate (Bergström et al., 1959) may limit the applicability of hippurate clearance in estimating RPF.

### 3.4 Intra‐renal transport of solutes

Using data from the nine patients with hippurate levels below 10 µM/ml in whom we could estimate GFR/RPF, we calculated the micromoles of solute that were filtered per ml plasma per minute and, by subtraction of that quantity from the AV difference, an estimate of what part of the AV difference was due to some combination of tubular secretion, reabsorption, or metabolism.

**Figure 4:**
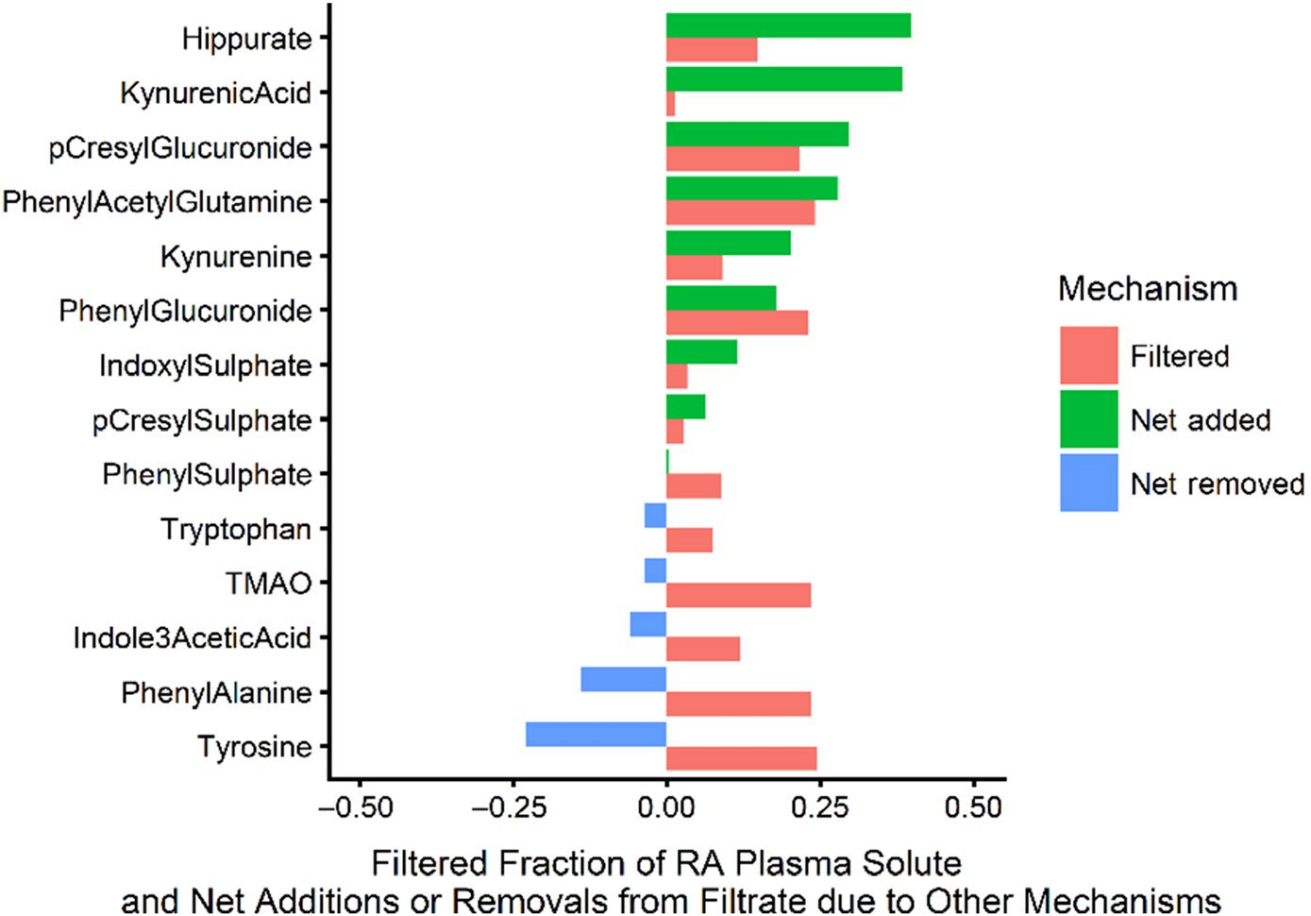
Glomerular filtration and net changes due to processes other than filtration

Positive values on the X‐axis of Figure 4, shown in red, represent the fraction of the Extraction Ratio filtered at the glomerulus. The values shown in green represent that additional fraction of the Extraction Ratio which is due to the net addition of solute to the tubular fluid. The negative values, shown in blue, represent the fraction by which the amount of solute filtered exceeded the Extraction Ratio difference and was returned to the plasma by reabsorption. As applied to hippurate excretion, for example, the mean RA concentration was 2.44 µmol/ml. An estimated 14% of the RA concentration was filtered, while an additional 54% was secreted. In total, 63% of RA hippurate was removed from the plasma. In addition to hippurate, kynurenic acid, phenylacetylglutamine, p‐cresyl glucuronide, phenyl glucuronide, indoxyl sulfate, and p‐cresyl sulfate were secreted. In contrast, 92% of filtered tyrosine was reabsorbed. Only 2% of filtered tyrosine remained in the tubular fluid to be excreted. Similarly, phenylalanine, tryptophan, phenyl sulfate, and indole 3 acetic acid were reabsorbed to varying degrees.

Kynurenine appears to be a special case. While the analysis of *A* − *V* difference (Figure 4) suggests that there was both filtration and secretion of this solute the levels of kynurenine in the urine and the very low ratio of kyurenine clearance to creatinine clearance (Figure 1) suggest reabsorption. We suggest that the explanation for this apparent discordance is the intrarenal conversion of kynurenine to kynurenic acid or other compounds. Our conclusion that kynurenine is secreted or metabolized is confirmed by the close agreement of our estimate of the ratio of RV and artery concentrations of this solute, 0.71 and the value of 0.70 obtained by Rhee et al. (2013, supplementary table 2).

In examining the application of hippurate clearance for the estimation of RPF, we compared our data with PAH and inulin clearances published previously by Bergström et al. (1959). Figure 3 illustrates the relationship between IVC concentration of hippuric acid and the ratio of its clearance to that of creatinine for the 12 subjects in this study in whom urine samples were collected. We take the close agreement of our estimates of RPF based on creatinine/hippurate values to estimates based on inulin/PAH values as confirmation of the validity of hippurate clearance as a measure of RPF.

## 4 DISCUSSION

The renal clearance of hippurate exceeded creatinine clearance in all subjects studied. Hippurate/creatinine clearance ratios >4 are close to the clearance of infused para‐aminohippurate relative to inulin clearance (Bergström et al., 1959) and support our view that hippurate clearance might provide a measure of ERPF. Observed ratios below 4.0 may reflect reduced RPF in some of the patients who were studied during evaluation of either hypertrophic cardiomyopathy or cardiac arrhythmias. Reduced cardiac output or other medications may have reduced renal blood flow. Bergström et al. (1959) reported the extraction ratio of p‐aminohippurate in 30 normal subjects and subjects with variable degrees of reduced GFR, as measured by inulin clearance. The extraction ratio for p‐aminohippurate in normal subjects averaged 0.905 ±0.19% a value quite close to that reported by Smith and Smith et al. (1945). The extraction ratio was found to range from 0.942 to 0.805 in 24 of 25 subjects diagnosed as having “essential hypertension”, but decreased RPF and increased FF, were noted in 12 of these subjects. The hippurate extraction across the renal vascular bed observed in this study lead us to believe that hippurate clearance can serve as a measure of ERPF in patients with levels of hippuric acid below 5 µM.

## 5 CONCLUSIONS

The study confirms the role of renal tubular secretion, presumed to be mediated by OAT 1 and possibly OAT 3, in the excretion of hippurate, kynurenic acid, indoxyl sulfate, p‐cresyl sulfate, phenyl acetyl glutamine, phenyl glucuronide, and p cresyl glucuronide. Observational studies have suggested that some of these protein‐bound solutes may play a major role in mediating the cardiovascular pathology that accounts for death after 3 years of hemodialysis (United States Renal Data System). Rhee et al. (2013) studying a panel of retention solutes associated with CKD in the Framingham cohort measured the arteriovenous gradient of these solutes in nine subjects with moderate reduction in glomerular filtration. Kynurenine, kynurenic acid, and indoxyl sulfate were identified as solutes transported by the renal tubule. None of the other solutes measured in our study was included in the platform employed in Rhee's study. The identification of uremic retention solutes that are generated by the gut microbiome actively secreted by renal tubular OAT transporters, and mediate toxic effects on vascular or other tissues, provides a possible basis for the development of modifications of protein binding or tubular transport that might correct features of the uremic syndrome.

This study provides experimental evidence that the clearance of hippurate, requiring only a timed urine collection and a single midpoint plasma sample from a peripheral vein, can provide a good estimate of ERPF. It is likely that measurement of the GFR, by the clearance of creatinine, and ERPF, by the clearance of hippurate, in a timed urine collection and a single blood sample, could yield important insights into the hemodynamic abnormalities that characterize conditions such as “cardiorenal syndrome” and the early phase of “acute kidney injury”.
